# 重视外科并发症对肿瘤患者生存的影响

**DOI:** 10.3779/j.issn.1009-3419.2018.03.25

**Published:** 2018-03-20

**Authors:** 剑华 傅

**Affiliations:** 中山大学肿瘤医院胸外科 Department of Thoracic Surgery, Cancer Hospital of Zhongshan University

肺癌术后呼吸道并发症：如痰液潴留、肺不张、肺炎、呼吸功能不全等。尤以年迈体弱者、原有缓慢支气管炎、肺气肿者发病率较高。对于肺癌术后并发症的预防在临床肺癌手术治疗中具有重要的意义。如果能通过对肺癌患者术前肺通气功能和弥散功能的检测，评估、预测术后并发症的发生，则可通过调整手术方案和术中、术后用药，加强围手术期的护理，达到降低术后心肺并发症发生的目的。

本文客观的回顾性分析了胸腔镜肺癌肺切除术后患者住院时间延长（ > 7天）的发生比例、病因及高危因素，并进行了生存分析，数据翔实，具有很强的可读性，可为肺外科提供宝贵的经验。如何缩短住院日、减少并发症的发生，是外科永恒的话题，术后并发症有些不可避免，有些却可通过术中、术后的恰当处理得以避免。肺漏气是肺切除术后的常见轻度并发症，通常是可通过细心、耐心的术中操作减少其发生的，建议文中能更加详细的介绍自己的经验和读者交流；术后肺不张，常见的原因是患者排痰不畅，如何能让患者在术后良好咳痰，是一个系统工程，包括：术前教育、指导性训练、术前术中肺保护以减少痰的产生、术后心理辅导鼓励其咳痰，甚至支气管镜吸痰，来避免其发生，希望作者能在全文中给出有益的经验。

对于高危因素的分析，应纳入同期无并发症的病例进行多因素分析方可得出可靠的结果。

不同并发症严重程度分级患者的生存分析，意义不大；对没有并发症与有并发症的患者进行生存比较，更加有意义。

